# Beneficial Antimicrobial Effect of the Addition of an Aminoglycoside to a β-Lactam Antibiotic in an *E. coli* Porcine Intensive Care Severe Sepsis Model

**DOI:** 10.1371/journal.pone.0090441

**Published:** 2014-02-28

**Authors:** Paul Skorup, Lisa Maudsdotter, Miklós Lipcsey, Markus Castegren, Anders Larsson, Ann-Beth Jonsson, Jan Sjölin

**Affiliations:** 1 Section of Infectious Diseases, Department of Medical Sciences, Uppsala University, Uppsala, Sweden; 2 Department of Molecular Biosciences, The Wenner-Gren Institute, Stockholm University, Stockholm, Sweden; 3 Section of Anesthesiology & Intensive Care, Department of Surgical Sciences, Uppsala University, Uppsala, Sweden; 4 Centre for Clinical Research Sörmland, Uppsala University, Uppsala, Sweden; 5 Section of Clinical Chemistry, Department of Medical Sciences, Uppsala University, Uppsala, Sweden; University of North Dakota, United States of America

## Abstract

This study aimed to determine whether the addition of an aminoglycoside to a ß-lactam antibiotic increases the antimicrobial effect during the early phase of Gram-negative severe sepsis/septic shock. A porcine model was selected that considered each animal’s individual blood bactericidal capacity. *Escherichia coli,* susceptible to both antibiotics, was given to healthy pigs intravenously during 3 h. At 2 h, the animals were randomized to a 20-min infusion with either cefuroxime alone (n = 9), a combination of cefuroxime+tobramycin (n = 9), or saline (control, n = 9). Blood samples were collected hourly for cultures and quantitative polymerase chain reaction (PCR). Bacterial growth in the organs after 6 h was chosen as the primary endpoint. A blood sample was obtained at baseline before start of bacterial infusion for *ex vivo* investigation of the blood bactericidal capacity. At 1 h after the administration of the antibiotics, a second blood sample was taken for *ex vivo* investigation of the antibiotic-induced blood killing activity. All animals developed severe sepsis/septic shock. Blood cultures and PCR rapidly became negative after completed bacterial infusion. Antibiotic-induced blood killing activity was significantly greater in the combination group than in the cefuroxime group (p<0.001). Growth of bacteria in the spleen was reduced in the two antibiotic groups compared with the controls (p<0.01); no difference was noted between the two antibiotic groups. Bacterial growth in the liver was significantly less in the combination group than in the cefuroxime group (p<0.05). High blood bactericidal capacity at baseline was associated with decreased growth in the blood and spleen (p<0.05). The addition of tobramycin to cefuroxime results in increased antibiotic-induced blood killing activity and less bacteria in the liver than cefuroxime alone. Individual blood bactericidal capacity may have a significant effect on antimicrobial outcome.

## Introduction

Bacterial infections may progress to severe sepsis and septic shock, conditions associated with high mortality rates (20–60%) [1]–[5]. Initial treatment of septic shock includes fluid resuscitation and adequate antibiotic treatment [6]–[8]. In the initial empiric treatment of these severe infections a combination of antibiotics is commonly used to ensure that the causative organism is covered by at least one active drug. However, the advantage of covering the probable pathogen or pathogens with more than one active agent has not been without controversy. Several meta-analyses have failed to demonstrate beneficial effects of combination therapy in severe sepsis and Gram-negative bacteremia [9], [10]. However, a more advanced meta-regression analysis has recently shown that antibiotic combination therapy improves survival and clinical response in patients with life-threatening infections, particularly in those with septic shock [11]. That study was followed by a large retrospective propensity-matched multicenter cohort study evaluating the therapeutic benefit of early combination therapy with at least two antibiotics with activity against the pathogen isolated in patients with septic shock. Significant beneficial effects were observed, especially for ß-lactam antibiotics in combination with aminoglycosides, fluoroquinolones or macrolides [12]. Proposed principal mechanisms have been a synergistic antibacterial effect or immunomodulatory activity. Fluoroquinolones and macrolides have been shown to have direct immunomodulatory effects [13], whereas this effect is less obvious for aminoglycosides [12], [14]. For the ß-lactam/aminoglycoside combination, a faster bacterial clearance may be a more likely mechanism.

Whereas a synergistic effect of the ß-lactam/aminoglycoside combination has extensively been demonstrated *in vitro* [15], [16], *in vivo* experimental data are more limited. The *in vivo* studies have focused on local infections and mainly with *Pseudomonas spp.,* which is a well-known difficult-to-treat organism and endpoints have usually been bacterial growth in organs after several days of treatment [17]–[19]. Increased rate of bacterial clearance in the blood and reduced growth in the organs during the early phase of treatment are probably crucial factors in the treatment of life-threatening severe sepsis and septic shock. To our knowledge, these phenomena have not been studied in either clinical studies or experimental settings.

The aim of this study was therefore to investigate whether the addition of an aminoglycoside to a ß-lactam antibiotic has an effect on the *in vivo* killing rate of a common Gram-negative pathogen during the early phase of treatment in a porcine intensive care model of severe sepsis/septic shock. Because genetic polymorphism leads to differences in the response to bacterial challenges [20], [21], a secondary aim was to analyze the effect of individual blood bactericidal capacity at baseline on subsequent bacterial growth in the blood and organs.

## Materials and Methods

### Animals and Ethic Statements

This study included 27 apparently healthy Swedish landrace-breed piglets of both sexes. The Animal Ethics Board in Uppsala, Sweden approved the experiment (permit number C 215/5). The piglets were handled in accordance with the Guide for the Care and Use of Laboratory Animals. All surgery was performed under general anesthesia and efforts were made to minimize suffering. Animals had water and food access *ad libitum* until 1 h before the experiment.

### Anesthesia and Preparations

Anesthesia and ventilation in this study were performed as described in detail elsewhere [22]. In brief, the animals were sedated before transportation and preparations and then mechanically ventilated under intravenous general anesthesia. Acetated Ringer’s solution was administered, resulting in a total fluid administration rate of 15 mL×kg^−1^×h^−1^. All preparations were done under aseptic conditions. The following blood vessels were catheterized: auricular peripheral veins, the superior caval vein, the pulmonary artery (Swan Ganz catheter) and a cervical artery. Using vesicostomy, a urine catheter was inserted into the bladder. After completed preparations, the animals were allowed 40 min of stabilization before initiation of the experiment, i.e. the baseline. A bolus of 4% succinylated gelatin solution 30 mL×kg^−1^ was given 45–60 min before baseline.

Initial respiratory settings were: respiratory rate 25 min^−1^, inspiratory-expiratory ratio 1∶2, inspired oxygen fraction (FiO_2_) 0.3, positive end-expiratory pressure (PEEP) 5 cm H_2_O and tidal volume 10 mL×kg^−1^. Tidal volume was adjusted before the start of the protocol to result in an arterial partial pressure of carbon dioxide (PaCO_2_) of 38–41 torr (5.0–5.5 kPa). The ventilator used was either a Servo 900C or a Servo I (Siemens-Elema, Stockholm, Sweden).

To resemble an intensive care setting the animals were treated in accordance with a protocol to maintain vital parameters within preset limits (Table 1). Briefly, arterial partial pressure of oxygen (PaO_2_) was maintained at >10 kPa, mean arterial pressure (MAP) at ≥60 mm Hg and cardiac index (CI) at ≥2 L×min^−1^×m^−2^.

**Table 1:** Interventions performed to maintain vital parameters within preset limits.

Parameter	Threshold values for intervention	Interventions
**PaO_2_**	<10 kPa first time	Increase FiO_2_ to 0.6
**PaO_2_**	<10 kPa thereafter	1. Increase FiO_2_ to the next level: 0.6→0.8→1.0
		AND
		2. Increase PEEP to the next level: 5→8→10→14 cmH_2_0
		AND
		3. Lung recruitment maneuver^a^
**PaO_2_**	>30 kPa	Decrease FiO_2_ to the next level: 1.0→0.8→0.6→0.3
**MAP and/or CI**	MAP<60 mm Hg and/or CI <2.0 L×min^−2^×m^−2^	Start norepinephrine infusion 0.07 µg×kg^−1^×min^−1^. If ongoing norepinephrine infusion, increase rate one step: 0.07→0.13→0.29→0.54 µg×kg^−1^×min^−1^
**MAP**	MAP = MPAP (at <1 h after baseline)	Single dose of 40 µg norepinephrine i.v.
**MAP**	MAP = MPAP (at >1 h after baseline)	1. Single dose of 20 µg norepinephrine i.v.
		AND
		2. Start norepinephrine infusion 0.07 µg×kg^−1^×min^−1^. If ongoing, increase rate one step: 0.07→0.13→0.29→0.54 µg×kg^−1^×min^−1^
		AND
		3. Fluid bolus of 10 mL×kg^−1^ with 4% succinylated gelatin in normal saline
		AND
		4. Increase FiO_2_ to the next level: 0.3→0.6→0.8→1.0
		AND
		5. Increase PEEP to the next level: 5→8→10→14 cmH_2_0
		AND
		6. Lung recruitment maneuver^a^
**MAP**	>100 mm Hg	If ongoing norepinephrine infusion, decrease rate one step: 0.54→0.29→0.13→0.07 µg×kg^−1^×min^−1^

PaO_2_ = arterial partial pressure of oxygen, FiO_2_ = inspired fraction of oxygen, PEEP = positive end expiratory pressure, MAP = mean arterial pressure, CI = cardiac index, MPAP = mean pulmonary arterial pressure.

a PEEP was increased stepwise until a peak pressure of 35 cm H_2_O was reached. At this point, an inspiratory hold was performed for 10 s. Thereafter, the PEEP was stepwise decreased to the PEEP defined by the protocol. If MAP decreased to the level of the MPAP, the recruitment maneuver was aborted.

### Organism

*Escherichia coli* (*E. coli*), a common Gram-negative pathogen, was chosen for this study. The strain B09–11822 employed is a clinical isolate obtained from a patient with bloodstream infection and septic shock. This strain is encapsulated and serum resistant (analyzed to be serotype O-rough:K1:H7 at Statens Seruminstitut, Copenhagen, Denmark). In *in vitro* pilot studies prior to this study this strain demonstrated continued growth in both serum and whole blood from pigs as well as humans (data not shown). Minimal inhibitory concentrations (MICs) for cefuroxime and tobramycin were 4 µg×mL^−1^ and 0.5 µg×mL^−1^, respectively (Etest; Biodisk, Solna, Sweden). The bacteria were grown to logarithmic growth phase before the experiment.

### Antibiotics

Cefuroxime was chosen as the ß-lactam antibiotic and purchased as Zinacef (GlaxoSmithKline, Solna, Sweden). The aminoglycoside chosen was tobramycin, which was purchased as Nebcina (Meda, Solna, Sweden). These antibiotics were preferred because their porcine pharmacokinetic profiles have been studied and shown to have similarities to those in humans [23]. The antibiotic doses selected are those commonly recommended as maximal doses for the treatment of human clinical sepsis.

### Experimental Design

**Figure 1:**
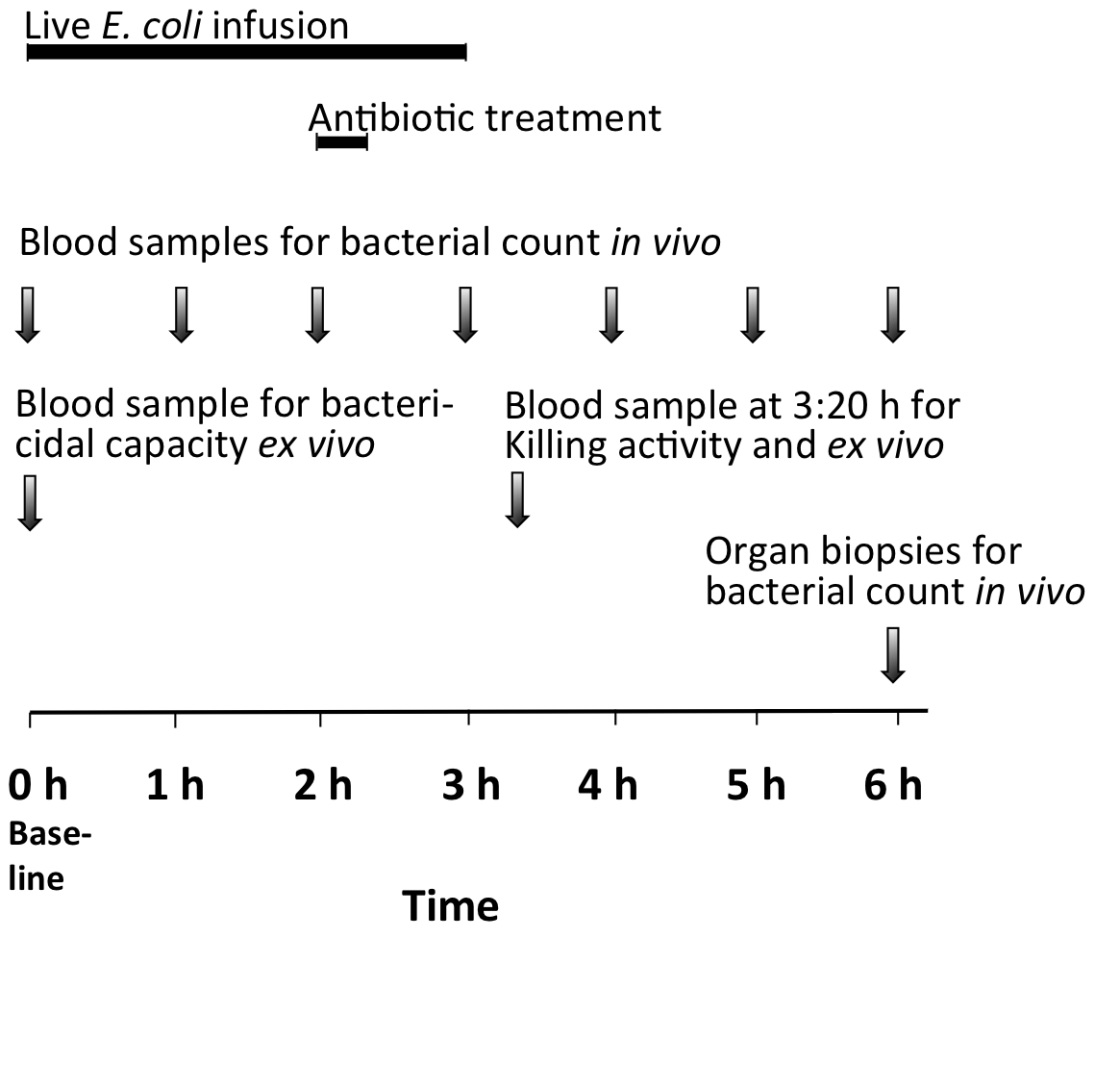
Experimental design. Black bars represent infusions while arrows indicate time points for sampling for bacterial analyses.

The experimental design of the study was prospective, parallel-grouped, randomized and placebo-controlled. In this non-lethal model an infusion of live *E. coli* of 5×10^8^ colony-forming units (CFUs) in 20 ml normal saline was administered at baseline via a central vein catheter at a constant infusion rate for 3 h (Figure 1 **)**. The number of bacteria of the infusate was controlled and the accepted range was 3.5–7.1×10^8^ CFU (8.70±0.15 log_10_ CFU). To secure that the bacteria remained in log phase the infusate was replaced hourly. Immediately before baseline, physiologic data were recorded and blood and urine samples obtained for laboratory analyses, including quantitative bacterial analysis. At baseline, the sealed envelope technique was applied to randomize the animals to one of three groups. The cefuroxime group received 750 mg cefuroxime in 50 ml saline plus 50 ml saline. The combination group received 750 mg cefuroxime in 50 ml saline plus 7 mg×kg^−1^ tobramycin in 50 ml saline simultaneously. The control group received placebo in the form of 100 ml saline alone. Antibiotics were given at 2 h as 20-min infusions in separate veins (Figure 1).

Hourly bacterial counts were taken from arterial blood samples (Figure 1). In addition, blood from three animals was analyzed for bacterial count at 15, 30 and 45 min after completion of the bacterial infusion.

Physiologic data were recorded and blood samples for the determination of hypoperfusion and organ dysfunction collected hourly. Blood samples for antibiotic concentrations were obtained at 3 h after baseline and then hourly. At 6 h after baseline, the animals were killed by a potassium chloride injection. Biopsies for bacterial count were obtained under aseptic conditions from standardized localizations in the spleen, right kidney and right lobe of the liver.

Arterial blood was collected for *ex vivo* experiments at 0 h to determine blood bactericidal capacity at baseline and at 3∶20 h for the antibiotic-induced activity in the blood after 1 h of treatment.

Starting at baseline, heart rate, MAP and mean pulmonary arterial pressure (MPAP) were continuously monitored and registered hourly. Cardiac output, assessed using the thermodilution method, and urine output were registered at the same time points. Calculations from registered values generated left ventricular stroke index (LVSWI) and CI using their regular formulas [24].

Arterial blood was analyzed for pH, gas tensions (PaO_2_, PaCO_2_), oxygen saturation and hemoglobin, using an ABL 5 and a Hemoximeter (Radiometer, Brønhøj, Denmark). Lactate concentration was analyzed by an i-STAT 1 (Abbott Scandinavia, Solna, Sweden). Urine and plasma creatinine analyses as well as plasma tobramycin were performed on an Architect Ci8200 analyzer (Abbott Scandinavia). Plasma concentration of cefuroxime was analyzed by reversed phase high-performance liquid chromatography and detected by mass spectrometry on an Agilent 1100 LC-MS (Agilent Technologies, Palo Alto, CA, USA).

### Determination of the Bacterial Killing Rate

#### In vitro bacterial killing

To compare the magnitude of the *in vivo* effects of the combination treatment the *in vitro* effect of the addition of tobramycin to cefuroxime was analyzed. Bacteria were resuspended to 10^7^ CFU×mL^−1^ in a Lysogeny broth (LB) medium containing cefuroxime (0 or 10 mg×L^−1^) or cefuroxime (10 mg×L^−1^)+tobramycin (20 mg×L^−1^) in 37°C. The pharmacodynamic parameter to predict efficacy for cephalosporins is concentration-independent and related to the time during which the drug concentration is greater than the MIC [25]. To achieve a maximal cephalosporin effect the cefuroxime concentration chosen was well above the MIC. The pharmacodynamic effect of aminoglycosides is associated with the peak concentration [26] and the tobramycin concentration chosen was in the magnitude of that expected clinically. Bacterial survival was determined after 0–6 h by plating serial dilutions on LB agar plates. Colonies were counted the following day. The detection limit was 10 CFU×mL^−1^. The experiment was performed in triplicate.

#### In vivo bacterial killing

Bacterial count in blood was determined hourly by plating 0.1 ml blood in triplicate (Figure 1). Organ biopsies of 1–2 g obtained at the end of the experiment were placed in 3 mL phosphate-buffered saline and homogenized for 4 min in a Stomacher 80 Biomaster (Seward Ltd, West Sussex, England) and 0.2 ml of each solution was plated in triplicate to determine viable count. The detection limit was 10 CFU×g^−1^.

#### Blood ex vivo bactericidal capacity of the animals at baseline

To determine the blood bactericidal capacity at baseline lithium-heparinized blood was obtained before infection and inoculated *ex vivo* with 10^5^ CFU×mL^−1^ *E. coli* in duplicate. Viable counts were plated after 0 and 6 h incubation at 37°C. The accepted range of the start inoculum was 5±0.15 log_10_ CFU×mL^−1^. The bactericidal capacity was assessed by the log_10_ count at 6 h.

#### Blood ex vivo killing activity of the animals after 1 h of treatment

Lithium-heparinized blood was drawn at 3∶20 h for determination of the *ex vivo* antibiotic-induced killing activity of the blood, 1 h after completion of the infusions of antibiotics or saline. The blood was inoculated with 10^5^ CFU×mL^−1^ *E. coli* in duplicate and incubated in 37°C. The accepted range of the inoculum was 5±0.15 log_10_ CFU×mL^−1^. Viable count was determined hourly.

### Quantitative PCR

DNA was extracted from 600 µl whole blood using a High Pure PCR template preparation kit (Roche diagnostics, Mannheim, Germany). To generate a standard curve different concentrations of *E. coli* B09–11822 (0–10^7^ CFU×mL^−1^) were added to uninfected whole blood from which DNA was extracted as described above. The detection limit of the assay was 10 CFU×mL^−1^. Primers fwd-(5′-tagcaaacgttctattggtgc-3′) and rev-(5′-catccagacgataagcatgagca-3′) directed against the K1 capsule gene were used as previously described [27]. Amplification was done in a 20 µl reaction mixture containing 10 µl template DNA and 75 nM of each primer in SYBR Green Master Mix (Roche diagnostics GmbH, Mannheim, Germany). Each reaction was run in duplicates. A touchdown PCR protocol was used to increase sensitivity. The following conditions were applied: an initial denaturation step at 95°C for 10 min, four cycles at 95°C for 10 s, 66°C for 30 s, 72°C for 1 min, followed by four cycles with an annealing temperature of 64°C, four cycles with an annealing temperature of 62°C and 40 cycles with an annealing temperature of 63°C. Bacterial DNA concentration was expressed as CFU equivalents×mL^−1^.

Three animals in each group were assigned for the addition of hourly quantitative PCR analysis of the arterial *in vivo* blood. From the *ex vivo* blood killing activity experiment, blood from three animals in each group was selected for quantitative PCR analysis.

### Calculations and Statistics

The difference in bacterial growth in the organs between the combination and cefuroxime regimen alone was chosen as primary endpoint. Because the logarithmic growth of bacteria approximates to normal distribution, Student’s *t* test was used in the comparison of bacterial growth. With nine animals in each group, a power of 0.8, an α-error of 0.05 and a standard deviation of 20%, the detectable difference was at least 30%. In the correlation analysis the Spearman rank order correlation was employed because the effect of the antibacterial treatment resulted in non-normally distributed organ bacterial counts when all three groups were combined. Statistica (StatSoft, Tulsa, OK, USA) was used for the statistical calculations and a p<0.05 was considered statistically significant.

## Results

### In vitro Bacterial Killing

**Figure 2:**
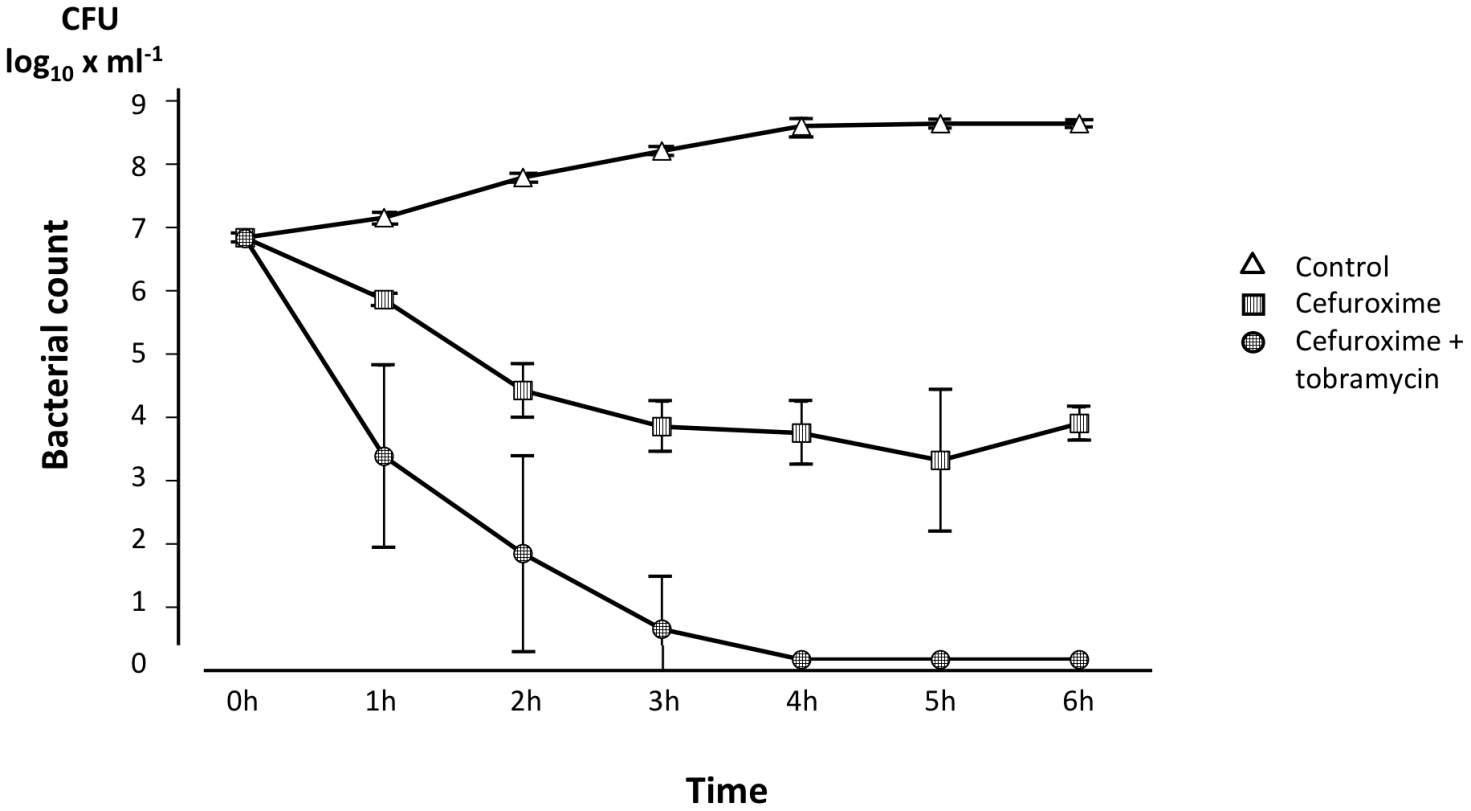
*In vitro* bacterial killing rate of the live *E. coli* strain used in the experiment. The bacteria were exposed to cefuroxime 10×L^−1^ alone, the combination of tobramycin 20 mg×L^−1^ and cefuroxime 10 mg×L^−1^ and control medium. The cefuroxime concentration chosen was well above the MIC, which should result in a maximal cephalosporin effect. The tobramycin concentration was >10 times the MIC and in the magnitude of that expected in clinical practice. Values are mean±SE (n = 3 for each treatment).

The bacterial killing rate *in vitro* is depicted in Figure 2. Maximal bactericidal activity was achieved after 5 h of cefuroxime exposure in a concentration well above MIC. The addition of tobramycin in the concentration of 20 mg×L^−1^ was found to reach similar bactericidal activity already after 1 h.

### Animal Experiment

After start of the bacterial infusion, all animals developed signs of severe sepsis/septic shock with deteriorated circulation as manifested by increases in MPAP, decreases in CI and the need for vasopressor support in 12 animals and respiratory interventions in 16. Hypoperfusion was demonstrated by a modest increase in arterial blood lactate of 38% and organ dysfunction by reductions in PaO_2_/FiO_2_, creatinine clearance and LVSWI of 41%, 53% and 38% of baseline values, respectively (Table 2). There were no noteworthy differences between the groups (data not shown).

**Table 2:** Physiological and laboratory parameters reflecting circulation, hypoperfusion and organ dysfunction (n = 27).

Time	MAP	CI	MPAP	Lactate	PaO_2_/FiO_2_	Creatinine clearance	LVSWI
	mm Hg	L×min^−1^×m^−2^	mm Hg	mmol×L^−1^	kPa	mL×min^−1^	g×m^−2^
0 h	91±11	5.7±0.9	21±4	1.6±0.7	61±8	150 (88–196)	56±11
1 h	87±16	4.0±1.0	35±6	1.7±0.7	59±10	165 (77–216)	41±12
2 h	94±15	3.4±0.9	36±7	2.0±1.0	52±12	75 (33–99)	40±13
3 h	93±18	2.9±0.6	40±7	2.2±1.0	41±12	96 (64–117)	34±13
4 h	97±14	3.0±0.5	34±8	1.8±0.5	39±12	77 (54–94)	37±8
5 h	92±17	3.3±0.7	33±8	1.5±0.6	40±15	61 (47–76)	36±8
6 h	87±19	3.5±0.7	32±9	1.3±0.5	36±16	70 (50–88)	35±11

Values are mean±SD, except creatinine clearance, which is given as median (interquartile range).

MAP = mean arterial pressure, CI = cardiac index, MPAP = mean pulmonary arterial pressure, PaO_2_ = arterial partial pressure of oxygen, FiO_2_ = inspired fraction of oxygen, LVSWI = left ventricular stroke work index.

Table 3 summarizes body weight, dose of infused bacteria, blood bactericidal capacity at baseline and *in vivo* blood bacterial count at 2 h, just before antibiotic treatment. No differences were observed between the groups in body weight or bacterial dose. A lower bactericidal activity of the blood was found, as manifested by a higher bacterial count at 6 h in the control group as compared with the other two groups (p = 0.08 and 0.03 vs. the cefuroxime and combination group, respectively). Bacterial concentrations at 2 h just before the start of the antibiotic treatment were also lower in the cefuroxime group compared with the combination (p = 0.01) and control (p = 0.003) groups. Bacterial count at 3 h was similar to that noted at 2 h and the same differences were seen (data not shown). Antibiotic concentrations are demonstrated in Table 4.

**Table 3:** Body weight, dose of infused *E. coli*, bactericidal capacity of the blood at baseline and *in vivo* blood bacterial count before start of antibiotic treatment at 2 h (mean±SD).

		All animals	Cefuroxime	Cefuroxime+tobramycin	Control
		n = 27	n = 9	n = 9	n = 9
Body weight, kg		24.8±2.7	24.9±2.0	24.6±4.3	25.0±1.2
Dose of infused bacteria, log_10_ CFU		8.72±0.05	8.71±0.06	8.72±0.05	8.71±0.04
Blood bactericidalcapacity at baseline	Bacterial count at 0 h, log_10_ CFU×ml^−1^	4.87±0.06	4.89±0.07	4.88±0.06^a^	4.84±0.04
	Bacterial count at 6 h, log_10_ CFU×ml^−1^	4.41±1.50	3.93±1.90	3.86±1.39^a^^,^ b	5.26±1.03
Blood bacterial count in vivo at 2 h, log_10_ CFU×ml^−1^		2.94±0.29	2.70±0.28^b^^,^ c	2.99±0.15^a^^,^ b	3.13±0.24

a n = 8. Start inoculum in one animal outside the accepted limits (see methods).

b significant vs. control group, p<0.05.

c significant vs. the combination group, p<0.05.

**Table 4:** Plasma concentration of cefuroxime and tobramycin (mean±SD).

Time	Cefuroxime		Tobramycin
	Cefuroxime group mg×L^−1^	Cefuroxime + tobramycin group mg×L^−1^	Cefuroxime + tobramycin group mg×L^−1^
	n = 9	n = 9	n = 9
3 h^a^	10.0±4.2	11.1±6.6	13.8±2.9
4 h	3.6±1.6	4.1±3.6	7.9±2.2
5 h	1.2±0.6	1.8±2.1	5.1±1.4
6 h	0.6±0.4	0.9±1.2	3.7±1.3

a time after baseline, i.e. 1 h after start of a 20-min infusion of antibiotics.

**Figure 3:**
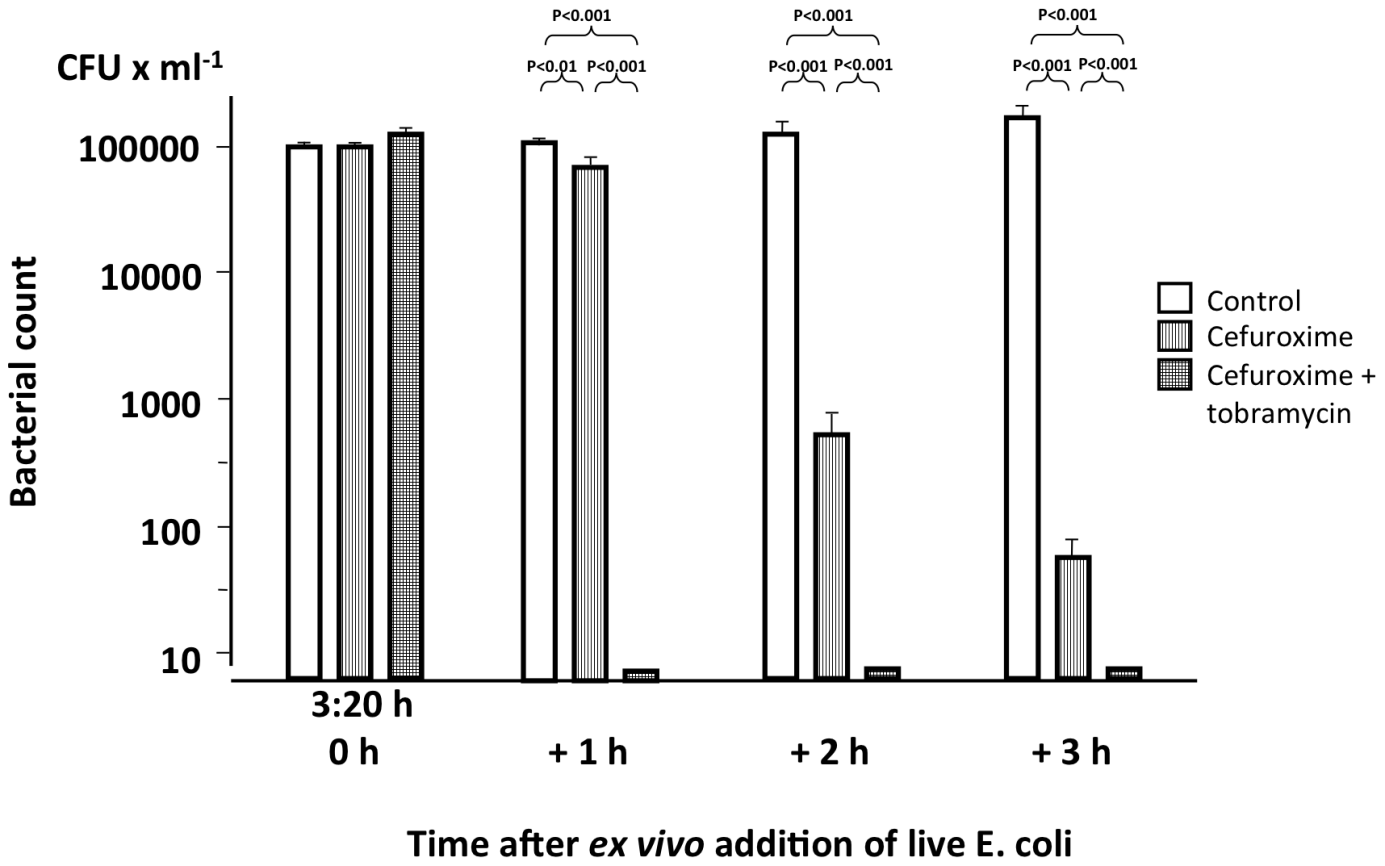
*Ex vivo* killing activity in blood obtained from treated animals. Bacterial count at various time points after the addition of live *E. coli* to a concentration of 1×10^5^ CFU×mL^−1^ to blood obtained from animals treated with cefuroxime alone or the combination of cefuroxime and tobramycin and from control animals at 3∶20 h, i.e. 1 h after completion of the administration of treatment. Values are mean±SE (n = 9 in each group).

After completion of the bacterial infusion, there was a rapid bacterial clearance in all groups, including the controls, with no live bacteria detectable at 15 min post-infusion or later. Thus, no differences in the bacterial killing rate could be detected. However, in blood sampled at 3∶20 h, i.e. 1 h after administration of antibiotics, the *ex vivo* blood killing activity in the animals treated with the combination of a ß-lactam antibiotic and an aminoglycoside was markedly bactericidal, whereas blood from cefuroxime-treated animals exhibited a more gradual killing (Figure 3).

**Figure 4:**
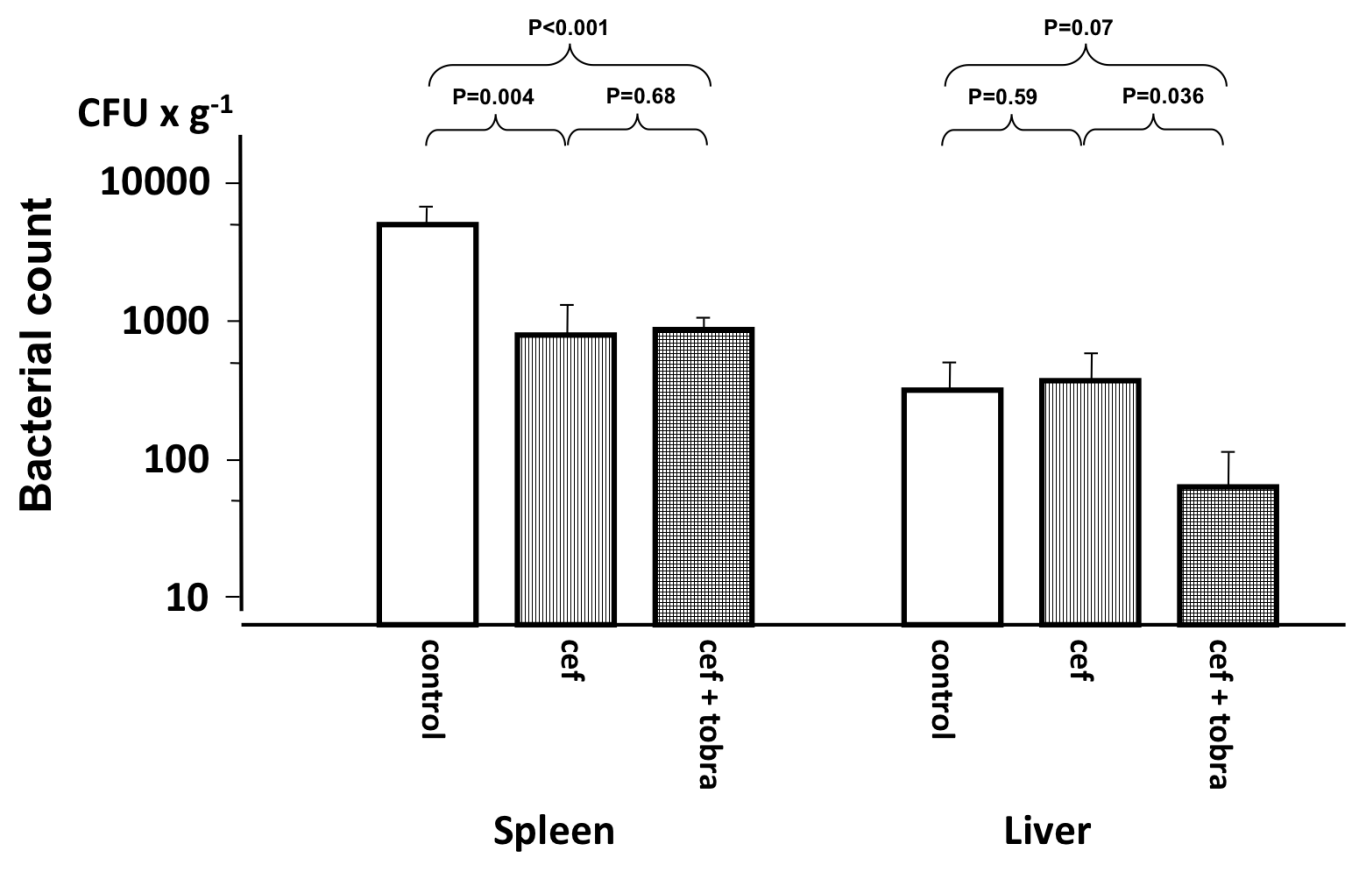
Bacterial count in the spleen and liver at the end of the experiment. Animals treated 4[61] or the combination of cefuroxime and tobramycin (cef+tobra) and control animals. Values are mean±SE (n = 9 in each group).

At the end of the experiment, growth of bacteria was seen in the liver and spleen; growth in the kidney was not detected in any of the animals. In the spleen both cefuroxime and the combination reduced growth significantly but there was no difference between the two groups (Figure 4). Growth of bacteria was lower in the liver than in the spleen (p<0.001 in the control group) and the combination regimen resulted in a significantly lower bacterial count than cefuroxime alone, which did not demonstrate any effect in comparison with the control group.

Bacterial growth in the organs was not significantly related to body weight or dose of infused bacteria within the dose limits (Table 5). In contrast, growth in the spleen was associated with blood bacterial count at 2 and 3 h and with the blood bactericidal capacity at baseline. Although there was a correlation to growth in the spleen, growth in the liver was not similarly linked to blood bacterial count and blood bactericidal capacity. However, if analysis were performed only in the control animals not exposed to antibiotics, correlation coefficients for blood bactericidal capacity and growth in the spleen and liver were −0.43 and −0.33, respectively. In addition, blood bacterial count at 2 and 3 h correlated negatively to blood bactericidal capacity at baseline (Table 5).

**Table 5:** Spearman rank correlation between bacterial growth in the organs and body weight, dose of infused bacteria, blood bactericidal capacity at baseline and blood bacterial count *in vivo* at 2 and 3 h.

	Bodyweight	Dose of infusedbacteria	Blood bac-tericidalcapacity at baseline	Blood bac-terialcount at 2 h	Blood bac-terialcount at 3 h	Growth in theliver
Growth in the spleen	−0.30	−0.04	−0.42*	0.45*	0.41*	0.40*
Growth in the liver	−0.21	−0.10	−0.28	−0.08	−0.00	–
Blood bac-terial countat 2 h	−0.31	0.10	−0.41*	–	0.94*	
Blood bac-terial countat 3 h	−0.36	0.10	−0.46*		–	

*denotes a correlation coefficient that is higher than that required for p<0.05.

**Figure 5:**
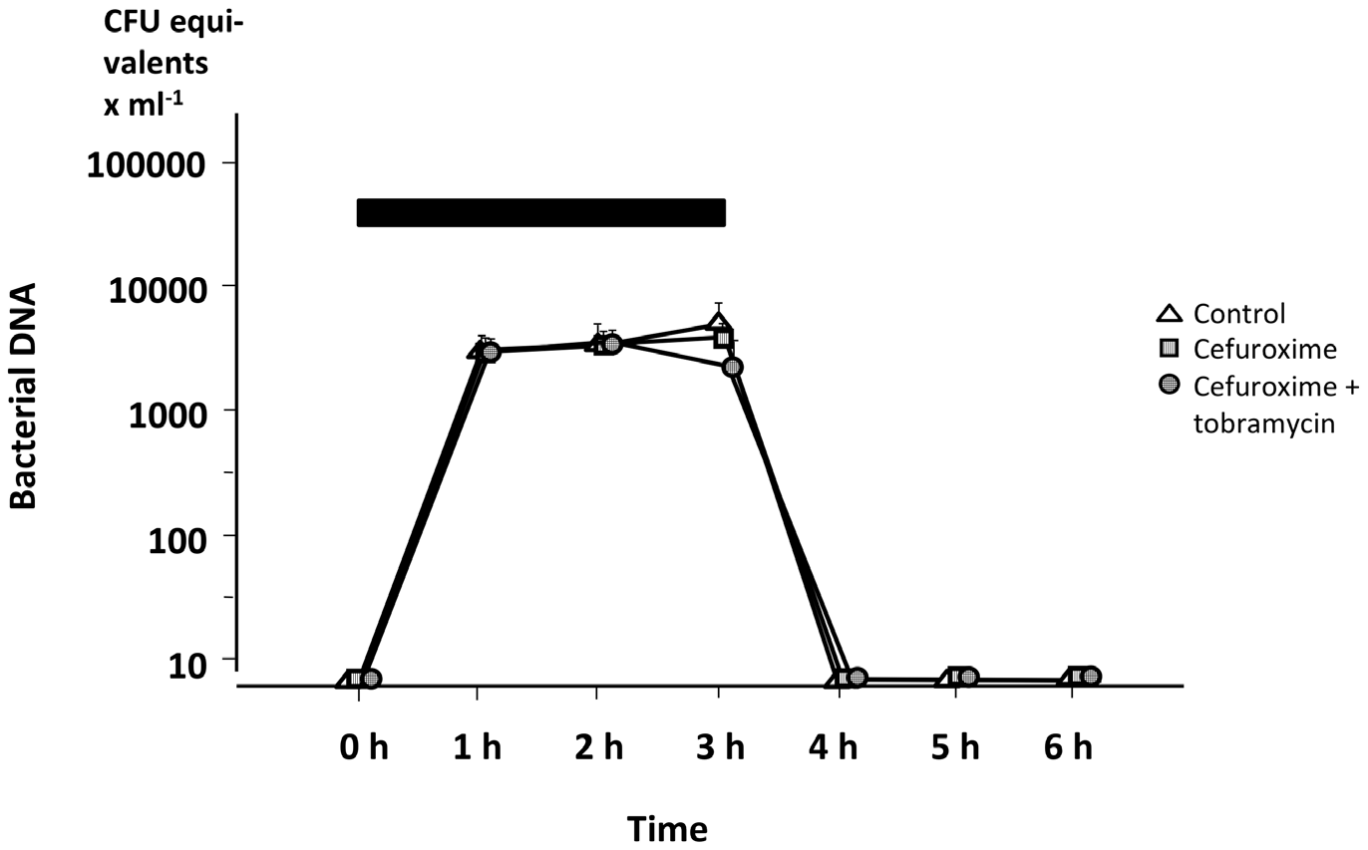
*In vivo* bacterial DNA in the blood of treated animals. The treatment was either cefuroxime alone, a combination of cefuroxime+tobramycin, or saline (control), during the 6 h experiment. Black bar denotes the infusion of live *E. coli.* The quantity of bacterial DNA is expressed as CFU equivalents. Values are mean±SE (n = 3 in each group).

**Figure 6:**
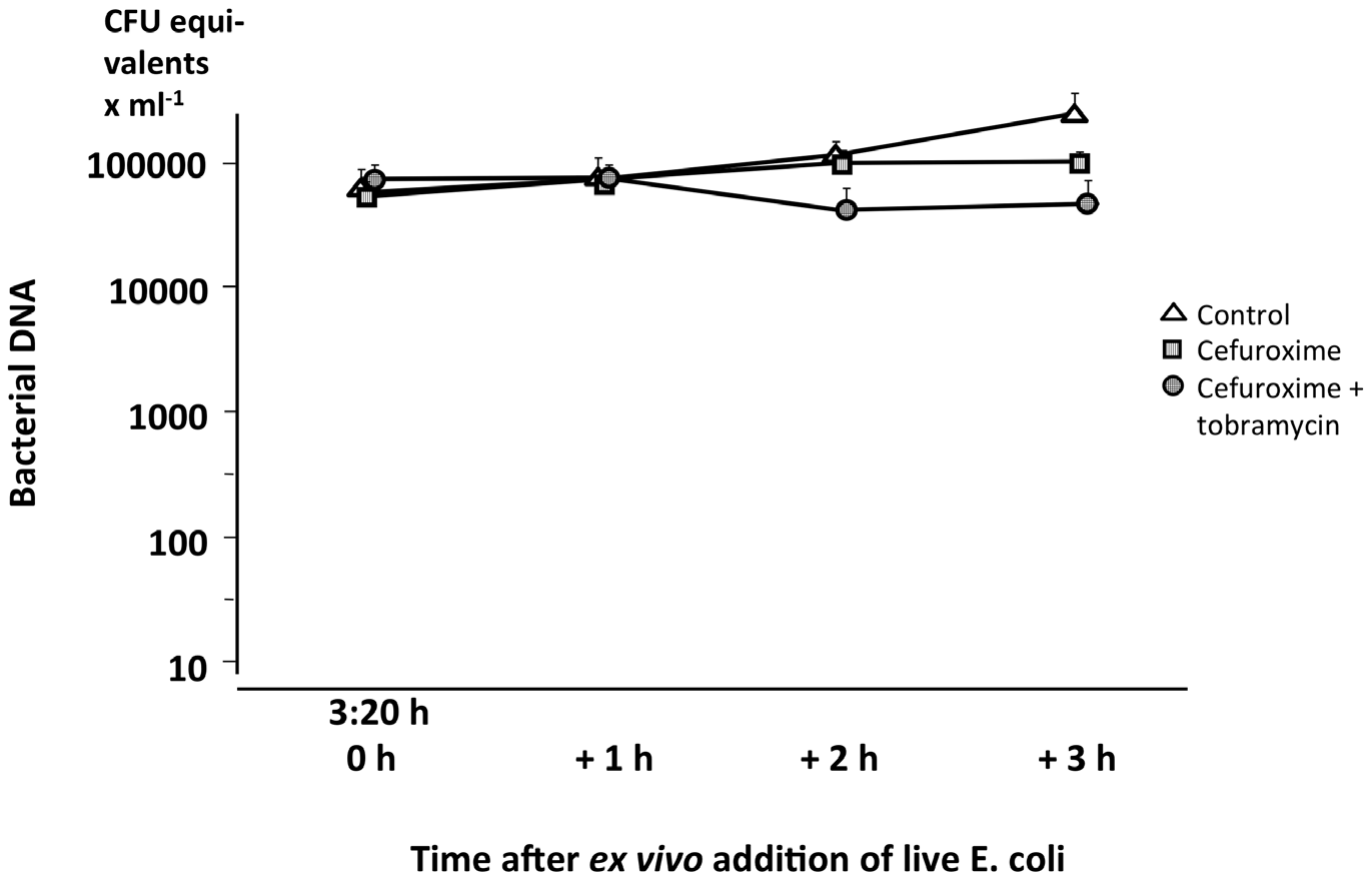
Bacterial DNA in the blood after *ex vivo* addition of live *E. coli*. Bacteria added to a concentration of 1×10^5^ CFU×mL^−1^ to blood obtained from animals treated with cefuroxime alone or the combination of cefuroxime and tobramycin and from control animals at 3∶20 h, i.e. 1 h after completion of the administration of treatment. The time points for sampling are the same as those in Figure 3, in which the antibiotic-induced killing activity significantly differed between the groups. The quantity of bacterial DNA is expressed as CFU equivalents. Values are mean±SE (n = 3 in each group).

As demonstrated in Figure 5, there was a rapid fall in bacterial DNA in arterial *in vivo* blood after completion of the infusion of bacteria, irrespective of treatment. No bacterial DNA was found in blood additionally sampled from two animals every 15 min during the first hour (data not shown). In contrast, in blood from the *ex vivo* killing activity experiments no similar bacterial DNA reduction was detected in any of the groups, not even in the combination group in which the bacterial killing rate was high (Figure 6).

## Discussion

The addition of an aminoglycoside to a ß-lactam antibiotic has been widely used for years in the treatment of severe sepsis and septic shock. However, its role has been controversial because of aminoglycoside-induced nephrotoxic and ototoxic side effects [28]–[31] and it is not clear whether the potential adverse reactions override the advantages of the combination [32], [33]. One area considered especially important to readdress is whether this combination confers benefits beyond broadening the antimicrobial spectrum [32]. In the presence of the difficulties to perform prospective randomized clinical trials in patients with these life-threatening intensive care unit (ICU) infections within the time frame available for initiation of antibiotic treatment [8], clinically relevant experimental studies are essential.

The model used in this study was designed to focus on the bacteriological consequences of bacteria that have entered the bloodstream during the early phase of severe sepsis/septic shock. In our pilot experiments there was a strong dose effect with a substantial mortality rate when the dose exceeded 10^9^ bacteria/3 h and therefore the variation of the dose of bacteria was kept at a minimum (Table 3). The intensive care protocol increases the clinical relevance of the study by adding the effects of sedation, mechanical ventilation and vasopressors on the innate immune response [34]–[38]. All animals developed severe sepsis with organ dysfunction necessitating intensive care. In pilot experiments biopsies from different tissues have been cultured and the only organs that demonstrated growth were the spleen and liver. In the final protocol kidneys were included as a control for sterility. Because several clinical studies have shown that treatment with an aminoglycoside alone results in worse outcome compared with treatment with ß-lactam antibiotics [39], [40], no tobramycin arm was included in the protocol. The cefuroxime concentration profile was consistent with that in patients with a sepsis-induced increase in extracellular volume and a slightly augmented renal clearance often observed during the initial phase in younger patients with activated systemic inflammatory response [41]. The tobramycin concentration was slightly lower than that reported from ICU patients but well above the pharmacodynamic target of a Cmax/MIC ratio of >10 [42], [43]. Thus, taken together, the concentrations achieved in the present model were clinically relevant to permit the same pharmacodynamics effects as those expected in patients with severe sepsis/septic shock after the first dose.

Additional to the bacterial dose, several factors may affect the number of bacteria in the blood at the time of treatment. In Table 5 a significant association to the blood bactericidal capacity at baseline and blood bacterial count at 2 h is demonstrated. Despite this and similar blood bactericidal capacities, there were a higher number of bacteria in the blood of the animals in the combination group than in the cefuroxime group at 2 h, the time when treatment was initiated (Table 3). Although standardized efforts were made to keep infused bacteria in the log phase and metabolically active, a difference in viability is probably the most likely source of explanation to this difference. However, even with a higher bacterial count at the start of antibiotic treatment, the addition of tobramycin resulted in significantly lower growth in the liver at the end of the experiment (Figure 4), indicating a beneficial antimicrobial effect of the combination treatment. In addition, combination treatment resulted in a considerably increased blood *ex vivo* killing activity (Figure 3).

An extremely rapid *in vivo* clearance of live bacteria from the blood with no relapse during the study period was observed in all animals, including the controls. The rapid bacterial clearance is further highlighted by the bacterial DNA analyses demonstrating that bacterial DNA was present in a nearly constant concentration after bacterial killing *ex vivo*, whereas none was detectable *in vivo* once the bacterial infusion had been completed (Figures 5 and 6). The implication of this finding is not clear but during the initial phase of severe sepsis both live and killed bacteria seemed to be rapidly eliminated from the circulation. This rapid elimination may help explain the surprisingly low sensitivity of bacterial PCR tests in suspected sepsis [44], [45]. The prompt elimination might have been caused by an intact and effective innate immune response in the otherwise healthy animals. In contrast, the elimination rate of both live bacteria and bacterial DNA might be slower in patients and experimental animals with more prolonged processes and increasing anti-inflammatory responses. However, this possibility needs further investigation.

Bacterial count in the liver was more than one log_10_ lower than in the spleen. At the end of the experiment, the bacteria are most probably located intracellularly in the phagocytic cells of the organs, where it is possible to escape from extracellularly distributed antibiotics. It may be speculated that the difference in bacterial count might be due to dissimilarities in the set up of these cells in the two organs. In the spleen bacteria leave the circulation in the red pulp and enter an open blood system without endothelial lining. In this compartment a large number of macrophages are residing that are well prepared for phagocytosis of bacteria before they pass through the endothelial slits of the splenic venous sinuses and re-enter the circulation [46], [47]. In the liver the Kupffer cells protrude into the lumen of the hepatic sinusoids and bacteria are mainly taken up directly from the circulating blood during bacteremia without any filtration or trapping mechanisms [48]. Thus, such a process may explain the lower bacterial uptake in the liver than in the spleen.

In the spleen cefuroxime alone and the combination both resulted in a significant decrease in bacterial count of similar magnitude, whereas in the liver the combination reduced bacterial count significantly more than cefuroxime alone (Figure 4). Free antibiotic concentrations in the extracellular fluid of different organs have been demonstrated to approximate those in plasma [49]. However, for cefuroxime alone to reach an identical killing activity as the combination, the bacteria have to be exposed for more than 3 h (Figure 3). Exposure to antibiotics in the liver must have been shorter to permit the superior blood *ex vivo* killing activity of the combination to play a role. Although we noted rapid elimination of bacteria from the blood, our data indicate that antibiotic exposure in the liver was long enough for the combination to exert its antimicrobial effect before the bacteria were fully phagocytized and inaccessible for antibiotics.

The blood killing activity *ex vivo* and the results from the liver indicate an antimicrobial advantage with the combination, whereas results from the spleen do not. Yet, it must be emphasized that growth in the spleen correlated significantly to the bacterial count in the blood before administration of treatment (Table 5) and that the higher amount of bacteria at this time point in the combination group (Table 3) might have masked a smaller difference in splenic bacterial growth in favor of the combination. Moreover, antimicrobial killing in the spleen might have occurred earlier in the combination group and, if so, it cannot be excluded that this might also be of some importance.

Several *in vitro* studies have demonstrated synergism between ß-lactam antibiotics and aminoglycosides [32], [50]. However, different host defense mechanisms play important roles and *in vitro* results are not always transferrable to the *in vivo* situation. In immunocompetent animal models previous studies have examined and compared single versus combination therapy of ß-lactam antibiotics and aminoglycosides for the treatment of Gram-negative bacterial infections in peritoneal infection [17], [51]–[53], pneumonia [54], [55], pyelonephritis [56] and endocarditis [57]–[59]. With few exceptions, these studies indicate that the combination of ß-lactam antibiotics and aminoglycosides results in improvements of either microbiological results or survival. Whereas these studies focus on prolonged treatment, the present study aims at empirical early treatment. In the absence of prospective randomized clinical trials the present findings are of clinical relevance and lend strong support to the results of the studies by Kumar et al [11], [12], indicating that the addition of an aminoglycoside to a ß-lactam antibiotic may result in beneficial antimicrobial effects in addition to merely broadening the antibacterial coverage.

This study has some potential limitations. The clot implantation technique of the bacteria into the peritoneum might have resulted in an even more clinical relevant model [60]. However, such a model first results in a local infection, which subsequently might be followed by a variable bacteremia. To maximally standardize the challenge of bacteria within the bloodstream and the development of severe sepsis/septic shock the intravenous administration route was chosen. Furthermore, in clinical practice cefuroxime has often been replaced by newer cephalosporins. Still, the pharmacodynamic effect on cephalosporin-susceptible bacteria is the same regardless of the cephalosporin used, and the reason for choosing cefuroxime was its known porcine pharmacokinetic profile with striking similarities to that in humans [23].

## Conclusion

Bacteria and gene fragments are rapidly eliminated from the circulation, regardless of the treatment. The beneficial pharmacodynamic effects of the combination of a ß-lactam antibiotic and an aminoglycoside seen *in vitro* were also observed in the early phase of severe sepsis and septic shock. Furthermore, the individual blood bactericidal effect may confound the results in this type of experiment and hence should be taken into account when interpreting the findings.
